# Supplementary material for: High Behavioral Reactivity to Novelty as a Susceptibility Factor for Memory and Anxiety Disorders in Streptozotocin-Induced Neuroinflammation as a Rat Model of Alzheimer’s Disease
Source: Int J Mol Sci. 2024 Oct 28;25(21):11562. doi: 10.3390/ijms252111562 (PMC11546707; doi:10.3390/ijms252111562)
Supplement: Supplementary file 1 [file ijms-25-11562-s001.zip › Supplementary the material Table S1.pdf]

Table S1. Behavioural activity in the elevated plus maze (EPM) during the Test and re-Test (60 min after the Test) measured as entries to the open/closed arms and centre (a) and time spent in the open/closed arms and centre (b) of the maze in rats with high (HR) or low (LR) reactivity to novelty at baseline (before injections) and 45 and 90 days after intracerebroventricular injections of streptozotocin (STZ, n=16) or citrate buffer (VEH, n=14).

a	Parameter/Group		Baseline		STZ 45		STZ 90		VEH 45		VEH 90	
			**HR**	**LR**	**HR**	**LR**	**HR**	**LR**	**HR**	**LR**	**HR**	**LR**
	**Open arm entries**	**Test**	2.5±1	1.4±0.55	0±0#	1.33±0.58	0±0	0±0	1.67±0.58&	3.67±1.53	0±0#	1.67±0.58
		**re-Test**	1.25±0.5	1±0	0±0	0.67±0.58	0±0#	1.33±0.58^	2±0#	0±0^	1±0#^	0±0^
	**Entries to the centre**	**Test**	3.75±1.71	5.6±2.61	5±0&	4.67±0.58	0±0#	1±0	3.67±1.15	3.67±1.53	2±1	4±1
		**re-Test**	9±3.27#^	4.2±1.3	0±0#^	1±0^	4.67±0.58^	4.67±0.58^	6±2	3.67±0.58	1.33±0.58	1.33±0.58^
	**Closed arm entries**	**Test**	4.75±1.71	6.6±2.61	6±0	5.67±0.58	1±0#	2±0	4.67±1.15	4.67±1.53	3±1	5±1
		**re-Test**	10.±3.27#^	5.2±1.3	1±0#^	2±0^	5.67±0.58^	5.67±0.58^	7±2	4.67±0.58	2.33±0.58	2.33±0.58^

b	Parameter/Group		Baseline	STZ 45	STZ 90	VEH 45	VEH 90
	**Time in the open arms (s)**	**Test**	12.33±6.90	3.65±3.20***@@	0±0@@@	20.88±4.9@	12±13.16
		**re-Test**	1.8±0.98^^^	0.6±0.66@	19.11±17.32^	20.38±19.09	2.5±2.74
	**Time in the centre (s)**	**Test**	67.53±23.76	46.64±15.97*&&&	7.5±8.80**@@@	31.23±10.54@@	31±13.22@@
		**re-Test**	52.56±18.63	9.01±10.63*&&@@@^^^	32.33±13.06@^^	22.34±6.48@@@^	27±11.28@@
	**Time in the closed arms (s)**	**Test**	220.13±25.44	249.67±16.88&&&@@	292.5±8.80**@@@	249.12±10.75&&@@	269±13.22@@
		**re-Test**	226.25±7.41^	281.18±18.27*&&@@^^	252.5±17.82*^^^	259.84±22.16	270.5±9.42@@

Explanations: Data are presented as mean ± SD and were analyzed using Mann–Whitney-*U* test; # - p≤0.05 indicates significance of differences between HR and LR within the STZ or VEH animals; ^ - p≤0.05, ^^ - p≤0.01, ^^^ - p≤0.001 indicate significance of differences between Test and re-Test; & - p≤0.05, && p≤0.01, &&& p≤0.001 indicate significance of differences between 45 day and 90 day after injection; @ - p≤0.05, @@ -p≤0.01, @@@ - p≤0.001 indicate significance of differences vs baseline.
