# Supplementary material for: High Behavioral Reactivity to Novelty as a Susceptibility Factor for Memory and Anxiety Disorders in Streptozotocin-Induced Neuroinflammation as a Rat Model of Alzheimer’s Disease
Source: Int J Mol Sci. 2024 Oct 28;25(21):11562. doi: 10.3390/ijms252111562 (PMC11546707; doi:10.3390/ijms252111562)
Supplement: Supplementary file 1 [file ijms-25-11562-s001.zip › Supplementary the material Table S2.pdf]

Table S2. Total numbers of leukocytes, percentage and total numbers of lymphocytes (TCD3^{+},TCD4^{+},TCD8^{+}, B, NK), granulocytes and monocytes, plasma level of interleukin 6 (IL-6) and interleukin 10 (IL-10), number of red blood cells (RBC), haemoglobin concentration (HGB), hematocrit (HCT), mean haemoglobin concentration in the red blood cell (MCHC), mean mass of the haemoglobin in the red blood cell (MCH), mean corpuscular volume (MCV), Red Cell Distribution Width (RDW) in rats non-divided and divided into rats with high (HR) or low (LR) reactivity to novelty 45 and 90 days after intracerebroventricular injections of streptozotocin (STZ, n=16) or citrate buffer (VEH, n=14).

Group/Parameter	STZ 45	STZ 90	VEH 45	VEH 90	STZ 45 HR	STZ 45 LR	STZ 90 HR	STZ 90 LR	VEH 45 HR	VEH 45 LR	VEH 90 HR	VEH 90 LR
**Leukocytes (No.x 10^{3}/μl)**	7.53±1.83	6.4±1.47	6.01±1.76	5.46±1.97	7.7±1.28	7.44±2.42	6.2±2.04	6.13±1.08	5.43±1.13	5.82±1.72	5.03±2.24	5.69±2.1
**Lymphocytes (No.x 10^{3}/ μl)**	5.71±1.77	4.78±1.4	4.46±1.37	4.63±1.81	6.3±1.44	5.38±2.2	4.83±1.7	4.45±1.44	4±1.21	4.42±1.36	5.48±2.44	4.27±1.51
**Lymphocyte percentage (%)**	77.48±5.22	75.03±10.02	76.33±6.37	76.49±5.96	75.82±6.08	78.72±4.63	72.58±13.56	72.58±13.56	74.68±7.43	78.61±4.36	75.38±4.46	76.5±7.15
**CD3^{+} cell percentage (%)**	38.08±7.21	36.57±7.78	31.34±10.41	35.63±5.22	32.9±6.78 $^{\#}$	43.27±1.84$^{\$}$	30.29±3.86	37.52±7.68	36.55±8.99	26.13±9.98	37.12±4.67	34.03±7.43
**CD3^{+}CD4^{+}CD8^{-} cell percentage (%)**	27.17±9.62$^{\&\&}$	16.42±3.53*	19.43±8.92	19.61±3.47	32.71±9.95$^{\$;\&}$	21.63±6.33	17.15±5.38	16.51±2.14$^{\$}$	15.63±6.09$^{\#}$	18.37±8.4	20.6±5.62	19.43±0.45
**CD3^{+} CD4^{-}CD8^{+} cell percentage (%)**	11.09±2 $^{\&}$	13.63±2.58*	9.35±3.63	11.42±1.46	12.26±2.23$^{\$}$	9.91±0.92$^{\&}$	12.35±2.91	15.03±2.47	6.16±3.06$^{\&}$	10.14±3.16	11.47±1.58	11.51±1.95
**CD3^{-}CD45RA^{+} cell percentage (%)**	11.47±2.55 $^{**;\&}$	17.53±4.23	11.18±5.26$^{\&\&}$	12.88±3.5	12.26±1.18	10.67±3.61	16.54±5.9	17.83±5	9.74±2.67	14.27±5.26	11.51±1.06	13.23±5.28
**CD3^{-}CD161a^{+} cell percentage (%)**	2±0.65	3.13±2.02	2.03±0.68$^{\&}$	1.4±0.51	1.99±0.33	2.02±0.98	1.92±1.48	3.3±2.27	2.01±0.8$^{\&}$	2.06±0.66	0.97±0.23$^{\#}$	1.9±0.23
**Granulocytes (No.x 10^{3}/ μl)**	0.87±0.31	0.74±0.35	0.83±0.16	0.68±0.31	0.6±0.2$^{\#}$	1.13±0.21$^{\$}$	0.5±0.1	0.8±0.34	0.85±0.17$^{\&}$	0.83±0.2	0.53±0.15	0.72±0.31
**Granulocyte percentage (%)**	8.39±3.77*	10.19±4.4	12.4±3.86	11.14±4.18	6.17±2.83$^{\$}$	9.43±4.41	7.27±1.33	12.03±5.24	13.6±1.49	12.47±4.69	10.48±5.06	12.53±3.22
**Monocytes (No.x 10^{3}/ μl)**	1±0.21**	0.89±0.43	0.57±0.24	0.73±0.32	0.8±0.1$^{\$\#}$	1.15±0.17$^{\$\$}$	0.6±0.2	1.03±0.55	0.43±0.06	0.57±0.23	0.93±0.37	0.64±0.28
**Monocyte percentage (%)**	13.6±4.06*	15.96±6.17	9.27±1.97$^{\&\&}$	13.13±3.23	11.7±2.66	14.66±5.02$^{\$}$	14.17±5.58	18.53±6.94	8.73±1.65$^{\&}$	9.5±2.16	14.15±3.3	12.47±3.52

Table S2 cont.

Group/Parameter	STZ 45	STZ 90	VEH 45	VEH 90	STZ 45 HR	STZ 45 LR	STZ 90 HR	STZ 90 LR	VEH 45 HR	VEH 45 LR	VEH 90 HR	VEH 90 LR
**Plasma IL-6 concentration (pg/ml)**	252.16±59.16	301.99±111.08	296.94±97.57	382.53±141.05	267.97±87.68	242.67±44.51	312.70±132.83	298.61±106.93	349.51±80.73$^{\&}$	266.89±98.55	362.33±149.28	396.34±148.26
**Plasma IL-10 concentration (pg/ml)**	36.29±10.22$^{\&}$	53.55±19.3*	35.5±9.6	35.05±15.32	41.29±8.29	36.03±10.4$^{\&}$	62.71±14.94	58±16.37$	33.52±8.55	36.49±10.71	41.47±15.74	32.92±14.66
**RBC (No. x10^{6}/μl)**	8.38±0.4$^{**\&}$	7.19±1.71	7.74±0.73	7.76±0.41	8.12±0.4$^{\#}$	8.57±0.28$^{\$\$}$	6.46±2.6	7.71±1.19	7.78±0.67	7.71±0.8	7.63±0.58	7.84±0.3
**HGB (g/dL)**	14.1±0.71*	13.85±0.76**	14.61±0.53$^{\&\&\&}$	12.82±0.83	13.72±0.74$^{\$\#}$	14.31±0.57	13.4±0.36$^{\#}$	14.4±0.64$^{\$}$	14.64±0.51$^{\&}$	14.7±0.44$^{\&\&\&}$	12.6±1.23	12.97±0.54
**HCT (%)**	44.55±2.38$^{\&}$	39.74±7.31	41.62±4.71	41.02±2.27	42.9±2.54$^{\#\#}$	45.7±1.46	35.8±11	43.23±3.25	41.73±4.38	41.45±5.38	40.4±2.76	41.4±2.18
**MCHC (g/dL)**	32.46±3.98$^{\&}$	33.18±1.14**	33.93±3.17$^{\&\&}$	31.22±1.49	34.74±4.39$^{\#}$	30.43±2.64 $^{\$\&\&}$	32.33±0.06	33.43±1.2$^{\$}$	33.71±3.07$^{\&}$	34.3±3.45	31.1±1.31	31.34±1.81
**MCH (pg)**	18.77+3.96	18.15±2.06**	18.59±1.67$^{\&\&\&}$	16.5±0.75	20.98±5.49	17.13±0.67$^{\$}$	17.1±0.3	19±2.78$^{\$}$	18.36±1.3$^{\&\&}$	18.33±1.3$^{\&\&}$	16.5±0.69	16.54±0.88
**MCV (μm3)**	55.71±3.08	56.5±6.52	54.23±0.86	52.77±1.42	56.43±3.81	55.31±2.59	57.67±8.14	56.75±6.89	54.08±2.53	53.65±2.19	53±1.22	52.71±1.7
**RDW (%)**	12.67±1.76$^{\&}$	14.94±1.73***	13.46±1.35$^{\&\&}$	12.42±0.84	11.7±2.14	12.96±1.61	14.8±1.99$^{\$}$	15.33±1.92$^{\$}$	12.83±0.31$^{\&}$	13.27±0.71	12.22±0.49	12.67±1.02

Group/Parameter	VEH 45 HR	VEH 45 LR	VEH 90 HR	VEH 90 LR
**$CD3^{+}$ cell (No.x 10^{3}/ μl)**	1.39±0.16	1.43±0.53	1.67±0.21	1.30±0.32
**$CD3^{+}CD4^{+}CD8^{-}$ cell (No.x 10^{3}/ μl)**	0.76±0.26$^{\&}$	1.02±0.48	0.85±0.04	0.96±0.26
**$CD3^{+}$ $CD4^{-}CD8^{+}$ cell (No.x 10^{3}/ μl)**	0.29±0.13	0.55±0.20	0.51±0.19	0.55±0.05
**$CD3^{-}CD45RA^{+}$ cell (No.x 10^{3}/ μl)**	0.58±0.31	0.59±0.4	0.52±0.08	0.52±0.24
**$CD3^{-}CD161a^{+}$ cell (No.x 10^{3}/ μl)**	0.79±0.04$^{\&}$	0.12±0.05	0.23±0.03$^{\#}$	0.54±0.06

Explanations: Data are presented as mean ± SD and were analyzed using Mann–Whitney-*U* test; # - p≤0.05, ## - p≤0.01 indicate significance of differences between HR and LR within the STZ or VEH animals; * - p≤0.05, ** - p≤0.01,*** p≤0.001 indicate significance of differences between STZ and VEH 45 and 90

days after injection; & - p≤0.05, && - p≤0.01, &&& p≤0.001 indicate significance of differences between 45 day and 90 day after injection; $ - p≤0.05, $$ - p≤0.01 indicate significance of differences between STZHR and VEHHR or STZLR and VEHLR.
